# The Association Between Adequate Prenatal Care and Severe Maternal Morbidity Among Teenage Pregnancies: A Population-Based Cohort Study

**DOI:** 10.3389/fpubh.2022.782143

**Published:** 2022-05-31

**Authors:** Jin Young Nam, Sarah Soyeon Oh, Eun-Cheol Park

**Affiliations:** ^1^Department of Healthcare Management, Eulji University, Seongnam, South Korea; ^2^Department of Social and Behavioral Sciences, Harvard TH Chan School of Public Health, Boston, MA, United States; ^3^Department of Preventive Medicine, Yonsei University College of Medicine, Seoul, South Korea; ^4^Institute of Health Services Research, Yonsei University College of Medicine, Seoul, South Korea

**Keywords:** severe maternal morbidity, prenatal care, Kessner Adequacy of Prenatal Care Index, teenage pregnancy, adolescent pregnancy, teenage mother, cohort study

## Abstract

**Background:**

The aim of this study was to examine whether inadequate prenatal care affect the risk of severe maternal morbidity in teenage pregnancies.

**Methods:**

We included 23,202 delivery cases among adolescent mothers aged between 13 and 19 years old with ≥ 37 weeks' gestational age. Data were derived from the National Health Insurance Service National Delivery Cohort in Korea between 2003 and 2018. We used a generalized estimating equation model while adjusting for numerous covariates to determine the adjusted relative risk (RR) associated with severe maternal morbidity. The main outcome measures were severe maternal morbidity and the Kessner Adequacy of Prenatal Care Index.

**Results:**

Severe maternal morbidity occurred in 723 (3.1%) of the 23,202 investigated delivery cases. The risk of severe maternal morbidity was 1.8-fold higher among adolescent mothers who had received inadequate prenatal care (RR, 1.81, 95% confidence interval [CI], 1.39–2.37) and 1.6-fold higher among those who had received intermediate prenatal care (RR, 1.59, 95% CI, 1.33–1.87) compared to those with adequate prenatal care. Synergistic effects of inadequate prenatal care and maternal comorbidities affected severe maternal morbidity.

**Conclusion:**

This study confirmed that inadequate prenatal care is associated with increased risk of severe maternal morbidity among pregnant teenagers. Notably, maternal comorbidity and inadequate prenatal care produced synergistic effects on severe maternal morbidity. Public health policy makers should focus on the development and implementation of programs to ensure that adequate prenatal care and financial/healthcare support is provided to teenage mothers during their pregnancies.

## Introduction

According to the World Health Organization, ~21 million teenage pregnancies occur each year, of which ~50% are unintended (1). Despite advances in most global regions to prevent pregnancies among adolescents, birth-related complications remain the leading cause of disability-adjusted life years and death among girls aged between 15 and 19 years (2). Previous studies have found that relative to women with average maternal age (20–24 years), teenage mothers face greater risk of eclampsia, endometritis, and systemic infections, while their babies face increased risk of low birth weight, preterm delivery, and intrauterine growth retardation (IUGR) (3). In low- and middle-income countries where young maternal age dramatically worsens the odds of child survival, teenage pregnancies may be associated with up to a 5.12-fold (95% CI: 2.85–9.20) increase in the risk of stillbirth, and 2.62-fold (95% CI: 2.22–3.08) increase in the risk of neonatal mortality (4). A combination of biological and social factors ranging from immature reproductive systems and low socioeconomic status (5), to unhealthy lifestyle behaviors (i.e., alcohol/tobacco misuse during pregnancy) (4), and unplanned pregnancy may affect the health of teenage mothers. Unplanned pregnancy will also place pregnant adolescents at greater risk than women of average maternal age with severe maternal morbidity (SMM) or maternal mortality (6).

SMM is defined as “an unintended outcome of the process of labor and delivery that results in significant short- or long-term consequences to a woman's health” (7). Increasingly, scholars and policy-makers are shifting the focus from the maternal mortality ratio (MMR) to SMM to measure obstetric quality and maternal wellness, as SMM is a potential precursor to maternal mortality and many more women experience life-threatening complications than die as a direct or indirect result of pregnancy (8). In the United States, ~11 to 16 of every 1,000 deliveries result in SMM, following a life-threatening diagnosis (e.g., acute myocardial infarction, eclampsia, adult respiratory distress syndrome, etc.) or lifesaving procedures (e.g., hysterectomy, blood transfusion, ventilation, etc.) during or within 42 days of giving birth (9), while ~211 of every 100,000 live births result in MMR (10).

Inadequate PNC has been associated with increased risk of SMM and prophylactic interventions responsible for increasing delivery medical costs in South Korea (11). While results suggest a clear association between teenage (below 19 years) or older (exceeding 35 years) maternal age and SMM, most research has focused on the risks of advanced maternal age which is a remarkably recent phenomenon among industrialized countries such as the United States and United Kingdom (12).

Furthermore, among studies targeting adolescent pregnancies and infant/maternal clinical health outcomes after adjusting for PNC, few have suggested a direct association between PNC and SMM among teenage mothers (13, 14), and there is limited evidence regarding the association of SMM with teenage pregnancy. Therefore, this study aimed to investigate whether adequate PNC relieves the risk of SMM in teenage pregnancies in South Korea after adjusting for potential confounding factors.

## Methods

### Data Source

Data from this population-based cohort study were retrieved from the National Health Information Database (NHID) between 2003 and 2013 and included data for utilization of healthcare, health screening, sociodemographic characteristics, and mortality for the whole South Korean population. These data were provided by the National Health Insurance Service (NHIS) which is the single insurer covering all Koreans (15). The NHID is composed of eligibility, national health screening, health care utilization, long-term care insurance and health care provider databases (15).

Data linkage in the NHI can be deterministically created using unique personal identification numbers assigned to residents of Korea by the government. In the NHID, de-identified join keys replacing the personal identifiers are used to interlink these databases and to secure ethical clearance (15). The health care utilization database is based on data collected during the process of claiming health care services, and includes information on records of inpatient and outpatient usage (diagnosis according to the *International Classification of Diseases, 10th Revision (ICD-10)*, length of stay, treatment costs, services received) and prescription. records (drug code, days prescribed, daily dosage) (15). The health care utilization database is the largest component of the NHID in data size. The health care provider database includes data regarding the types of health care institutions, health care human resources and equipment, covering all health care institutions in Korea (15).

The NHIS delivery cohort were extracted using the NHID claims database, which included data on all delivery cases in South Korea. We defined diagnosis and procedure codes for pregnancies by women aged between 13 and 19 years, who were hospitalized for delivery purposes and were enrolled for at least 280 days before childbirth through up to 6 weeks following childbirth between January 1, 2003 and November 19, 2018. Childbirth was identified as any inpatient hospital admission record including a pregnancy-related diagnosis (16) or procedure code for vaginal or cesarean delivery among women whose pregnancies had reached more than 37 weeks gestational age. We excluded teenage mothers who died during delivery hospitalization, those who were admitted for longer than 42 days, and those for whom the delivery hospital was unknown. A total of 23,202 delivery cases between 2003 and 2018 were included in this study.

### Severe Maternal Morbidity

SMM was defined according to SMM indicators and the possession of at least one of the 21 previously established ICD-10 diagnosis and procedure codes during the delivery hospitalization, using an algorithm developed by the Centers for Disease Control and Prevention (16). The original algorithm of 25 SMM indicators based on the 9th Revision of the ICD was published in 2012 (17); however, the United States transitioned to the ICD-10 diagnoses and procedures codes in October 2015. The updated algorithm identified 21 indicators of SMM that represented either serious complications of pregnancy or delivery, such as eclampsia or acute renal failure, or procedures used to manage serious conditions, such as a blood transfusion or hysterectomy. Of the 21 indicators, 16 were defined using diagnostic codes and 5 “used procedure” codes.

### Adequacy of Prenatal Care

We identified adequacy of prenatal care according to the Kessner Adequacy of Prenatal Care Index (KAPCI) (18). To meet an adequacy of the KAPCI rating, a woman must begin prenatal care in the first trimester and have nine prenatal care visits for a normal-length pregnancy (18). Inadequacy of the KAPCI rating was defined by a late start of prenatal visit during the third trimester, with four or less prenatal care visits until 34 weeks 7 days of pregnancy, while others were defined as intermediate KAPCI (18). In this study, gestation date and trimester period were evaluated from the childbirth date as the NHIS database did not include the starting date for gestation. The gestation date was estimated to be 280 days before the date of delivery. The trimester period was divided such that the first trimester was from gestation commencement up to 14 weeks and 0 days of pregnancy, the second trimester was from 14 weeks and 1 day to 28 weeks and 0 days, and the third trimester was from 28 weeks and 1 day to delivery date.

### Covariates

We selected covariates based on previous literature as follows: maternal socioeconomic characteristics included maternal age (17 years old or younger, 18, and 19 years old), household income level (quintile), type of insurance (self-employed insured, employee insured, and medical aid), residential area (city and rural), and employment status (employed and unemployed). Clinical variables included mode of delivery (spontaneous vaginal delivery, instrumental delivery, and cesarean section delivery), parity (1, 2, and 3 or more), twin birth status (singleton and twin birth), and maternal comorbidities (0, or 1 and more) using Howell's study (19). The type of hospitals was divided by the number of beds (beds <30, 30 ≤ beds <100, 100 ≤ beds <500, and beds ≥ 500), and the calendar year of childbirth.

### Statistical Analysis

We estimated the distribution of the general characteristics of our study population, for deliveries occurring between 2003 and 2018. The association between adequacy of prenatal care and SMM during delivery hospitalization following childbirth were the estimated adjusted risk ratio (RR) and 95% confidence intervals (CI) using a Poisson regression model with a robust error variance adjusted for all covariates. The Poisson regression model was generated for combined effect between adequacy of prenatal care and maternal comorbidities on SMM and evaluated for interactions. All statistical analyses were performed using SAS 9.4 (SAS Institute, Inc., Cary, NC, USA). The level of significance was set at *p* <0.05.

## Results

Table 1 shows the general characteristics of our sample according to related age groups. Of the 23,202 teenage pregnancies included in this study, 723 (3.1%) experienced an episode of SMM during the delivery hospitalization period. 11.1% of SMM events and 9.7% of non-SMM events were preceded by inadequate prenatal care.

**Table 1:** Baseline characteristics of study population with and without severe maternal morbidity.

	**No. (%)**				
	**No SMM** **(*****n** **=*** **22,478)**		**SMM** **(*****n** **=*** **723)**		**Total** **(*****n** **=*** **23,202)**
**Adequacy of prenatal care**					
Adequate	10,975	(48.8)	284	(39.3)	11,259
Intermediate	9,333	(41.5)	359	(49.7)	9,692
Inadequate	2,171	(9.7)	80	(11.1)	2,251
**Maternal age, year**					
≤ 17	4,083	(22.2)	159	(28.2)	4,242
18	5,180	(23.0)	187	(25.9)	5,367
19	13,216	(58.8)	377	(52.1)	13,593
**Household income level**					
Q1	8,801	(39.2)	291	(40.3)	9,092
Q2	6,200	(27.6)	210	(29.1)	6,410
Q3	4,824	(21.5)	142	(19.6)	4,966
Q$	2,654	(11.8)	80	(11.1)	2,734
**Type of insurance**					
Self-employed insured	11,694	(52.0)	377	(52.1)	12,071
Employee	7,847	(34.9)	239	(33.1)	8,086
Medical aid	2,938	(13.1)	107	(14.8)	3,045
**Residential area**					
City	7,609	(33.8)	242	(33.5)	7,851
Rural	14,870	(66.2)	481	(66.5)	15,351
**Mode of delivery**					
Spontaneous vaginal delivery	9,708	(43.2)	252	(34.9)	9,960
Instrumental delivery	7,956	(35.4)	195	(27.0)	8,151
Cesarean section delivery	4,815	(21.4)	276	(38.2)	5,091
**Parity**					
1	20,761	(92.4)	658	(91.0)	21,419
2+	1,718	(7.6)	65	(9.0)	1,783
**Status of multiple birth**					
Singleton	22,393	(99.6)	709	(98.1)	23,102
Multiple	86	(0.4)	14	(1.9)	100
**Maternal comorbidity**					
0	17,076	(76.0)	450	(62.2)	17,526
1+	5,403	(24.0)	273	(37.8)	5,676
**Type of hospital (No. of beds)**					
<30	923	(4.1)	123	(17.0)	1,046
30–99	2,294	(10.2)	175	(24.2)	2,469
100–499	6,776	(30.1)	185	(25.6)	6,961
500+	12,486	(55.6)	240	(33.2)	12,726
**Delivery year**					
2002	1,260	(5.6)	30	(4.2)	1,290
2003	1,312	(5.8)	41	(5.7)	1,353
2004	1,355	(6.0)	42	(5.8)	1,397
2005	1,264	(5.6)	42	(5.8)	1,306
2006	1,469	(6.5)	54	(7.5)	1,523
2007	1,734	(7.7)	50	(6.9)	1,784
2008	1,417	(6.3)	38	(5.3)	1,455
2009	1,508	(6.7)	41	(5.7)	1,549
2010	1,624	(7.2)	57	(7.9)	1,681
2011	1,727	(7.7)	50	(6.9)	1,777
2012	1,559	(6.9)	58	(8.0)	1,617
2013	1,450	(6.5)	44	(6.1)	1,494
2014	1,348	(6.0)	44	(6.1)	1,392
2015	1,060	(4.7)	40	(5.5)	1,100
2016	1,007	(4.5)	32	(4.4)	1,039
2017	761	(3.4)	35	(4.8)	796
2018	624	(2.8)	25	(3.5)	649

Table 2 presents the association between SMM and the risk factors adjusted for all covariates. Teenage mothers with intermediate or inadequate prenatal care had higher risk of SMM compared to those with adequate prenatal care (intermediate: RR 1.58, 95% CI 1.83–2.57; inadequate: RR 1.82, 95% CI 1.39–2.37). Teenage mothers who had cesarean section delivery (RR 2.17, 95% CI 1.83–2.57), those who had multiple birth (RR 2.07, 95% CI 1.27–3.36), and those who had maternal comorbidities (RR 1.81, 95% CI 1.55–2.12) had higher risk of SMM compared to reference group.

**Table 2:** Relative risk for the relationship between prenatal care and SMM among teenage pregnancy.

	**SMM**	
	**RR**	**(95% CI)**
**Adequacy of prenatal care**		
Adequate	1.00	
Intermediate	1.58	(1.33–1.87)
Inadequate	1.82	(1.39–2.37)
**Maternal age, year**		
≤ 17	1.00	
18	1.16	(0.97–1.40)
19	1.15	(0.97–1.36)
**Household income level**		
Q1	1.05	(0.81–1.36)
Q2	1.17	(0.91–1.50)
Q3	1.01	(0.77–1.32)
Q$	1.00	
**Type of insurance**		
Self-employed insured	1.03	(0.88–1.21)
Employee	1.00	
Medical aid	1.05	(0.82–1.34)
**Residential area**		
City	1.00	
Rural	1.07	(0.92–1.25)
**Mode of delivery**		
Spontaneous vaginal delivery	1.00	
Instrumental delivery	1.04	(0.86–1.25)
Cesarean section delivery	2.17	(1.83–2.57)
**Parity**		
0	1.00	
1+	1.24	(0.97–1.59)
**Status of multiple birth**		
Singleton	1.00	
Multiple	2.07	(1.27–3.36)
**Maternal comorbidity**		
0	1.00	
1+	1.81	(1.55–2.12)
**Type of hospital (No. of beds)**		
≥500	5.19	(4.19–6.43)
100–499	3.38	(2.79–4.09)
30–99	1.37	(1.13–1.66)
<30	1.00	
**Delivery year**		
2002	1.00	
2003	1.28	(0.82–2.01)
2004	1.52	(0.97–2.38)
2005	1.57	(1.00–2.48)
2006	1.75	(1.14–2.70)
2007	1.59	(1.02–2.48)
2008	1.39	(0.87–2.23)
2009	1.51	(0.95–2.40)
2010	1.92	(1.24–2.99)
2011	1.56	(0.99–2.45)
2012	2.04	(1.32–3.14)
2013	1.58	(0.99–2.50)
2014	1.70	(1.07–2.72)
2015	1.85	(1.15–2.97)
2016	1.43	(0.87–2.36)
2017	2.02	(1.24–3.29)
2018	1.61	(0.95–2.73)

**Figure 1:**
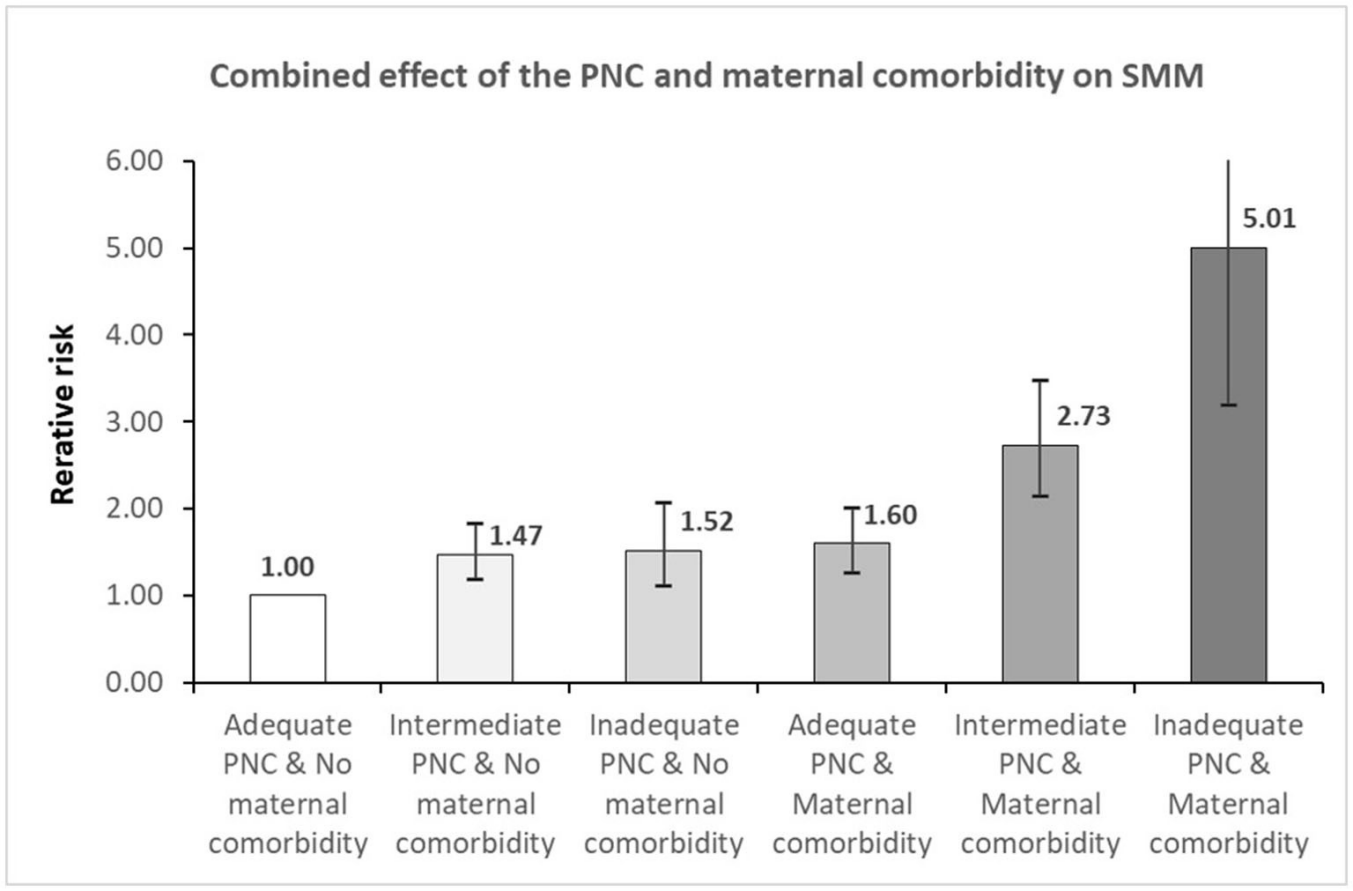
The combination effect of the relationship between prenatal care and maternal comorbidity on severe maternal morbidity. PNC, prenatal care; SMM, severe maternal morbidity. Adjusted for maternal age, household income, type of insurance, residential area, mode of delivery, adequacy of prenatal care, parity, status of multiple birth, maternal comorbidity, type of hospital, and delivery year.

We also examined the combined effects of prenatal care and maternal comorbidities with SMM, as shown in Figure 1 and Supplementary Table 1. Among teenage women, the highest risk of SMM occurred in those who had maternal comorbidities and had received inadequate prenatal care, with an ~5-fold increase in the risk of SMM among those without maternal comorbidities and adequate prenatal care (RR 5.01, 95% CI 3.19–7.87) (see, Supplementary Table 1). Moreover, women who had maternal comorbidities and intermediate prenatal care were at 2.7-fold higher risk of SMM compared to the reference group (RR 2.73, 95% CI 2.14–3.48). A synergistic effect of inadequate prenatal care and maternal comorbidities on SMM was observed.

## Discussion

### Main Findings

This nationwide population-based cohort study revealed that within a 16-year observational period, teenage mothers who had received inadequate prenatal care and those with maternal comorbidities may have increased risk of SMM compared to their counterparts. Interestingly, our findings also confirmed that inadequate prenatal care and maternal comorbidities could synergistically affect the risk of SMM among teenage mothers.

To our knowledge, this was the first study to examine the effects of PNC and SMM in teenage pregnancies; hence, the available evidence does not suffice for accurate comparisons. However, in a South Korean epidemiologic study of general pregnancy outcomes and prenatal care utilization among teenagers and adult women, 14.4% of teenage mothers reported to never having received PNC, which was in line with our findings (11.1% among those with SMM events; 9.7% among those with no SMM events) (20).

Similar to most studies focusing on the maternal age effect and its association with maternal complications, our study also found that teenage mothers are at greater risk of complications such as IUGR, preterm birth (21), anemia, oligohydramnios, failed labor induction, eclampsia, puerperal endometritis, systemic infections (22), and neonatal health outcomes including low birth weight (23), stillbirth, low Apgar score (24), severe neonatal conditions, and neonatal mortality compared to adults maternity, although in high-income countries.

While immaturity of the uterine or cervical blood supply in teenage mothers have been found to raise the risk of subclinical infection and prostaglandin production resulting in increased risk of preterm delivery (25), our investigation did not include the neonatal population or data related to preterm birth, so we were unable to confirm this association. However, we did find that ~37.8% of teenage mothers with SMM events had at least one maternal comorbidity, which was in line with the findings of a previous South Korean study where 38.1% of nulliparous women experienced maternal comorbidities (26).

### Interpretation

Several studies have highlighted the importance of prenatal care in reducing the risk of adverse maternal and neonatal outcomes. Many studies have proven the relationship between prenatal care and prevention of adverse maternal health outcomes such as eclampsia and puerperal infection (27), severe maternal morbidity and maternal mortality (28). However, these studies mostly focused on a combination of both adolescents and adults mothers.

Through our results, we inferred that limited access to prenatal care and lack of prevention and treatment interventions for adverse health conditions during adolescent pregnancy will also result in increased risk of eclampsia, puerperal endometritis, and systematic infection (27). Here, it was found that adolescents receive fewer benefits for prenatal care than that of adults, resulting in a gradual decline in the number of visits because of lack of awareness regarding available community services and the importance of early/regular care (13). It is probable that adolescents might prefer to conceal their pregnancies believing that they are ineligible for prenatal care services (13).

In particular, in South Korea, the entire population is covered by the National Health Insurance; hence, access to healthcare utilization could be adequate; however, emotional barriers to visit the obstetricians, and related mental illnesses such as depression may make it difficult for teenage mothers to receive PNC. A recent study, in high-income countries, demonstrated that over half of young women have experienced a mental disorder before becoming a parent (29), and that the prevalence of major depression and dysthymia associated with pregnancy at ages 10–35 years gradually increases through to the mid-twenties (30). In South Korea where enthusiasm for education is high, open discussions on adolescent sexual behavior have traditionally been taboo and the number of teenage abortions much greater than that reflected in official statistics (31). Although the government is rapidly shifting to increasing public awareness over adolescent sexual activities to prevent teenage pregnancies via education and protect the health of adolescent mothers and their babies (32), considerable stigma still exists.

In this study, we observed a synergistic effect of prenatal care and maternal comorbidities on SMM. Teenage mothers who had received inadequate prenatal care and had maternal comorbidities were at the highest risk of SMM compared to those who had adequate prenatal care and no maternal comorbidity. The result suggested that when adolescent mothers who did not know their adverse maternal conditions due to absence of prenatal care gave birth, it could result in multiple synergistic severe health outcomes, which could have been a preventable event. Since 2011, the Korean government caters for the medical expenses related to teenage pregnancies and has gradually expanded this support program (33). This pregnancy and childbirth medical expenses program gives financial support of approximately USD 1,000, which can cater for the medical expenses of all obstetric institutions, such as prenatal examination, childbirth, premature birth, natural miscarriage, delivery, prescription, and drug/treatment materials (33). However, to receive the financial support, one must have a pregnancy certificate from an obstetrician and have access to a bank, as the funds are accessed via a credit card for Boucher (33); these requirements may reduce access to PNC. In addition, the stigmatization of teenage pregnancies in South Korea is associated with psychological and emotional burden among the teen mothers, and this delays or hinders PNC visits. Fortunately, the rate of teenage pregnancies for those aged **≤** 15 years has gradually decreased until recently; ~0.17% reduction in total pregnancies was observed from 2015 to 2020 (34). However, in 2014, the total medical cost for teenage pregnancies was higher than that for pregnancies of mothers aged 20 to 24 years (35), underscoring the need for an enhanced support program for teenage pregnancies to improve health, as well as boost access to financial and medical resources. Therefore, the problem of obstetric accessibility, financial and legal regulation, stigmas, and lack of social support for adolescent pregnancy should be corroborated.

Second, although our finding did not reveal such results, substance abuse including smoking, alcohol consumption, and other drugs are escalated to a higher risk of use and dependence in early adulthood (36). Maternal smoking is linked to adverse birth and child outcomes, such as low birth weight, stillbirth, and sudden infant death (37). Heavy alcohol drinking during perinatal period is also predictive of adverse birth outcomes (30). Studies using animal models have indicated that even low levels of drinking during the first trimester affect craniofacial development (38). Commonly, women attempt to modify their substance use behaviors during their late pregnancy for fetal development; however, considering that ~40% of pregnancies are unintended and awareness generally occurs around 6 to 8 weeks of gestation, it is likely that many teenage mothers in our population also engaged in such activities (38). For Korean adolescents, academic stress caused by underachievement has been associated with increased risk of tobacco and alcohol use (39), while sexual relations after drinking have been found to increase the odds of teenage pregnancy by an OR of 25.1 among female adolescents (40). Such factors indicate that further studies are needed to develop an effective education program for preventing adolescent pregnancy and reducing delinquent behavior among adolescents.

Third, obesity in women has increased rapidly during adolescence and young adulthood across all countries (30). Obesity during pregnancy may lead to pre-eclampsia, induced labor, emergency cesarean section delivery, postpartum hemorrhage, and preterm delivery (41). In a study on teenage maternal obesity and its effect on perinatal outcomes, it was also found that compared with normal-weight teenagers, obese teenagers are at increased risk of can deliveries (adjusted OR: 4.3, 95% CI: 2.4-7.6) and gestational diabetes (adjusted OR 4.2, 95% CI: 1.5–12.1) (42). Therefore, obesity and derived adverse conditions should be managed via adequate prenatal care in teenage pregnancy to prevent adverse outcomes.

### Strengths and Limitations

This study has several limitations. First, this study could have residual confounding variables, which are not included in the information in the NHIS database, such as marital status, educational level, body mass index, and maternal lifestyle habits including drinking or smoking, etc., which may be associated with SMM. Although the database has no information on body mass index, we attempted to diagnosis codes using the ICD-10 for obesity (E66). Furthermore, as this database did not include gestational age, we could not control for this factor. However, we attempted to calculate the prediction of gestational age from the childbirth date to confirm the adequacy of prenatal care. Second, in order to calculate gestational age more precisely, we excluded teenage mothers who had preterm birth. In fact, preterm birth is a well-known risk factor of adverse maternal health outcomes, especially of teenage mothers. However, this database has diagnosis codes for preterm birth (childbirth <37 weeks) but there is no information on the exact gestational age, which might reflect a significantly different health status for both the mother and fetus during under 37 weeks; therefore, we excluded preterm birth teenage mother to ensure homogeneity. Another limitation is the heterogeneity of the study population. In this study, teenage pregnancy was defined as pregnancy in girls aged 13–19 years, but the study population had different characteristics, such as physical maturity, socioeconomic status, or marital status. Therefore, further research should determine whether each age group has a different effect on the relationship between comorbidity and prenatal care with regard to SMM.

Nevertheless, this study has several strengths. First, to our knowledge, this was the first study of teenage pregnancy on SMM using large population-based long-term cohort study. Most of previous studies included adolescent and adults (young or all) (5, 43); however, this study included only adolescent girls aged between 13 and 19 years, which might result in homogeneity of the study population. Second, this was the first study to examine the combined effect of the adequacy of prenatal care and maternal comorbidities on SMM. Finally, our study confirmed that primary prevention efforts are necessary for adolescent pregnancies, even among the South Korean populations where the rate of teenage pregnancies is only 0.49% (20), because there is still a statistically significant association between young maternal age and SMM.

## Conclusions

Our study confirmed that inadequate prenatal care may be associated with increased risk of SMM among pregnant teenagers. Notably, maternal comorbidities and inadequate prenatal care produced synergistic effects on SMM. Therefore, public health policy makers should focus on the development and implementation of programs to ensure adequate prenatal care and support financial accessibility of healthcare to improve the health of teenage mothers.

## Data Availability Statement

The datasets presented in this article are not readily available because the data were obtained from the database of the National Health Insurance Sharing Service. The authors are not eligible to duplicate and disseminate the database. Requests to access the datasets should be directed to https://nhiss.nhis.or.kr/bd/ab/bdaba000eng.do.

## Ethics Statement

This study design was reviewed and approved by the Institutional Review Board of Eulji University (No. EU21-005). Written informed consent from the participants' legal guardian/next of kin was not required to participate in this study in accordance with the national legislation and the institutional requirements.

## Author Contributions

JN and SO: conceptualization and writing-original draft. JN: data curation. SO and E-CP: validation. JN: resources and supervision. All authors: investigation, methodology, writing, reviewing, and editing. All authors contributed to the article and approved the submitted version.

## Funding

The authors disclosed receipt of the following financial support for the research, authorship and/or publication or this article: This research was supported by Basic Science Research Program through the National Research Foundation of Korea (NRF) funded by the Ministry of Science, ICT & Future Planning (2020R1C1C1013668).

## Conflict of Interest

The authors declare that the research was conducted in the absence of any commercial or financial relationships that could be construed as a potential conflict of interest.

## Publisher's Note

All claims expressed in this article are solely those of the authors and do not necessarily represent those of their affiliated organizations, or those of the publisher, the editors and the reviewers. Any product that may be evaluated in this article, or claim that may be made by its manufacturer, is not guaranteed or endorsed by the publisher.
