# Supplementary material for: The Association Between Adequate Prenatal Care and Severe Maternal Morbidity Among Teenage Pregnancies: A Population-Based Cohort Study
Source: Front Public Health. 2022 May 31;10:782143. doi: 10.3389/fpubh.2022.782143 (PMC9192951; doi:10.3389/fpubh.2022.782143)
Supplement: Supplementary file 1 [file Table_1.DOCX]

**Supplementary table 1. The combination effect of the relationship between prenatal care and maternal comorbidity on severe maternal morbidity**

|  | **SMM** | | |
| --- | --- | --- | --- |
|  | **RR** | **(95% CI)** | |
| Combination of PNC and maternal comorbidity |  |  |  |
| Adequate PNC & No maternal comorbidity | 1.00 |  |  |
| Intermediate PNC & No maternal comorbidity | 1.47 | (1.19 - | 1.83) |
| Inadequate PNC & No maternal comorbidity | 1.52 | (1.11 - | 2.07) |
| Adequate PNC & Maternal comorbidity | 1.60 | (1.27 - | 2.01) |
| Intermediate PNC & Maternal comorbidity | 2.73 | (2.14 - | 3.48) |
| Inadequate PNC & Maternal comorbidity | 5.01 | (3.19 - | 7.87) |

PNC: prenatal care; SMM: severe maternal morbidity

Adjusted for maternal age, household income, type of insurance, residential area, mode of delivery, adequacy of prenatal care, parity, status of multiple birth, maternal comorbidity, type of hospital, and delivery year.
